# Enhancing natriuretic peptide signaling in adipose tissue, but not in muscle, protects against diet-induced obesity and insulin resistance

**DOI:** 10.1126/scisignal.aam6870

**Published:** 2017-07-25

**Authors:** Wei Wu, Fubiao Shi, Dianxin Liu, Ryan P. Ceddia, Robert Gaffin, Wan Wei, Huafeng Fang, E. Douglas Lewandowski, Sheila Collins

**Affiliations:** 1 Integrative Metabolism Program, Sanford Burnham Prebys Medical Discovery Institute at Lake Nona, Orlando, FL 32827, USA.; 2 Division of Endocrinology and Metabolism, Huashan Hospital, Fudan University, Shanghai 200040, China.; 3 Cardiovascular Metabolism Program, Sanford Burnham Prebys Medical Discovery Institute at Lake Nona, Orlando, FL 32827, USA.

## Abstract

In addition to controlling blood pressure, cardiac natriuretic peptides (NPs) can stimulate lipolysis in adipocytes and promote the “browning” of white adipose tissue. NPs may also increase the oxidative capacity of skeletal muscle. To unravel the contribution of NP-stimulated metabolism in adipose tissue compared to that in muscle in vivo, we generated mice with tissue-specific deletion of the NP clearance receptor, NPRC, in adipose tissue (*Nprc*^*AKO*^) or in skeletal muscle (*Nprc*^*MKO*^). We showed that, similar to *Nprc* null mice, *Nprc*^*AKO*^ mice, but not *Nprc*^*MKO*^ mice, were resistant to obesity induced by a high-fat diet. *Nprc*^*AKO*^ mice exhibited increased energy expenditure, improved insulin sensitivity, and increased glucose uptake into brown fat. These mice were also protected from diet-induced hepatic steatosis and visceral fat inflammation. These findings support the conclusion that NPRC in adipose tissue is a critical regulator of energy metabolism and suggest that inhibiting this receptor may be an important avenue to explore for combating metabolic disease.

## INTRODUCTION

Obesity, a result of calorie intake exceeding energy expenditure, is frequently associated with tissue inflammation and insulin resistance. It is a major risk factor for many metabolic disorders, including type 2 diabetes (T2D), cardiovascular diseases, and several types of cancer (1–3). The link between obesity and T2D depends largely on the activity and function of adipose tissue (4). In mammals, there are two major types of adipose tissue with distinct physiological roles. White adipose tissue (WAT) stores excess nutrients in the form of triglycerides, and brown adipose tissue (BAT) dissipates heat through an uncoupling protein 1 (UCP1)–dependent mechanism. The “rediscovery” of functional BAT in healthy adult humans (5) and the identification of UCP1-positive brown-like (beige) adipocytes in white adipose suggests that UCP1 is a potential target for increasing energy expenditure to control body weight (6–9).

The cardiac-derived natriuretic peptides (NPs), atrial NP (ANP), and the related B-type NP (BNP) are key factors that control blood pressure by acting on the kidney (10). The physiological actions of ANP and BNP are mediated by binding to NP receptor A (NPRA), which activates its guanylyl cyclase domain to produce cyclic guanosine 3′,5′-monophosphate (cGMP), leading to activation of cGMP-dependent protein kinases [protein kinase G (PKG)] (11, 12). The other receptor for these cardiac NPs, NP receptor C (NPRC), functions to clear the NPs from circulation through receptor-mediated internalization (13). Thus, the ability of the NPs to elicit a biological response depends on the relative ratio of the functional receptor NPRA to the “clearance receptor” NPRC.

NP receptors are present in adipose tissue (14). ANP stimulates lipolysis in cultured human adipocytes, with potency similar to the β-adrenergic agonist isoproterenol (15). We have shown that NPs can also induce the adipose “browning” program through the same NPRA-cGMP-PKG signaling cascade (16). We have previously shown that ANP treatment of cultured human adipocytes increases mitochondrial biogenesis, UCP1 abundance, and uncoupled respiration (16). Clinical studies in humans report that NPs can increase energy expenditure and fat oxidation independent of the β-adrenergic axis (17). Compared with lean individuals, obese subjects have increased NPRC abundance in adipose tissues, resulting in a decreased NPRA/NPRC ratio and blunted cellular responses (18–20). This increase in NPRC in adipose tissue also has been posited to contribute to the lower circulating BNP concentration observed in obese subjects, possibly due to increased peptide uptake (21).

The ability of NPs to stimulate lipolysis is reported to be primate-specific and not to occur in rodent adipose tissues (22). This species difference is likely due to the fact that NPRC abundance in the rodent is 100-fold higher (22, 23). In line with this hypothesis, primary adipocytes from *Nprc* null mice (*Nprc*^*−/−*^) show a lipolytic response to ANP (16). In addition, *Nprc*^*−/−*^ mice have markedly reduced body weight and fat mass as well as higher amounts of UCP1 in their white adipose depots. We therefore proposed that NPRC plays a critical role in modulating the metabolic effect of NPs, particularly in the adipose tissue (16). However, because studies in rodents and humans suggest that NPs may be involved in skeletal muscle metabolism (24–27) and whole-body *Nprc*^*−/−*^ mice also exhibit a skeletal overgrowth phenotype (23, 28), it is unclear whether adipose tissue is the major site by which NPs increase energy expenditure. Here, we showed that deletion of *Nprc* in adipose tissue (*Nprc*^*AKO*^), but not in skeletal muscle (*NprcMKO*), protected against diet-induced obesity and insulin resistance. *Nprc*^*AKO*^ mice exhibited increased energy expenditure, reduced inflammation, and a redistribution of lipid storage from liver to visceral fat. These findings suggest that the adipose tissue is the major site of NP-mediated metabolic effects in mice lacking NPRC under high-fat diet (HFD)–feeding conditions.

## RESULTS

### HFD-fed *Nprc*^*−/−*^ mice were leaner and exhibited improved glucose tolerance

**Fig. 1.**
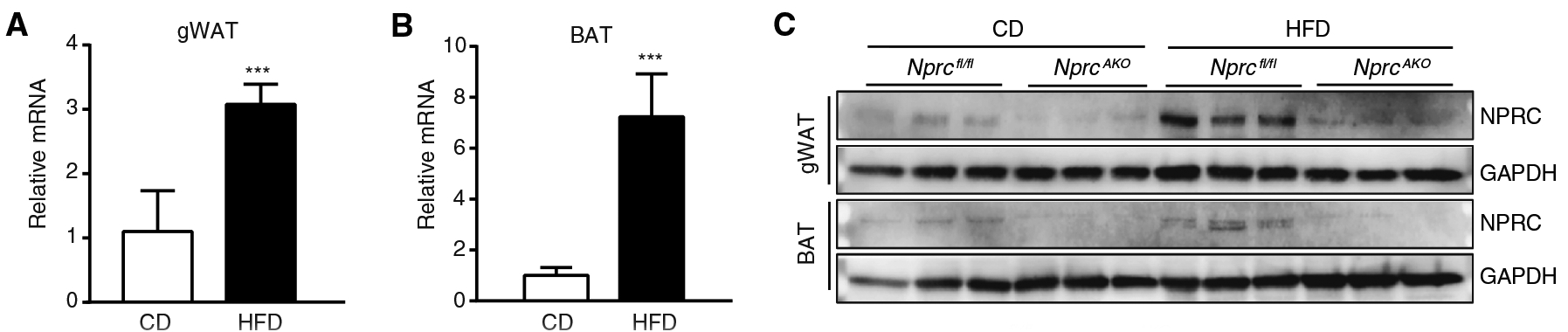
*Nprc* is increased at the mRNA and protein levels in adipose tissue by HFD feeding. (**A** and **B**) *Nprc*^*fl/fl*^ and *Nprc*^*AKO*^ mice were fed with a chow diet (CD) or an HFD for 12 weeks. qRT-PCR for the expression of *Nprc* mRNA relative to *36B4* in gonadal WAT (gWAT) (A) and BAT (B) of CD-fed (*n* = 3) and HFD-fed (*n* = 5) *Nprc*^*fl/fl*^ mice. (**C**) Western blotting analysis for NPRC protein performed on the lysates from gWAT and BAT of *Nprc*^*AKO*^ and *Nprc*^*fl/fl*^ mice. Blots are representative of three separate cohorts. ***P < 0.001, unpaired two-tailed Student’s *t* test.

**Fig. 2.**
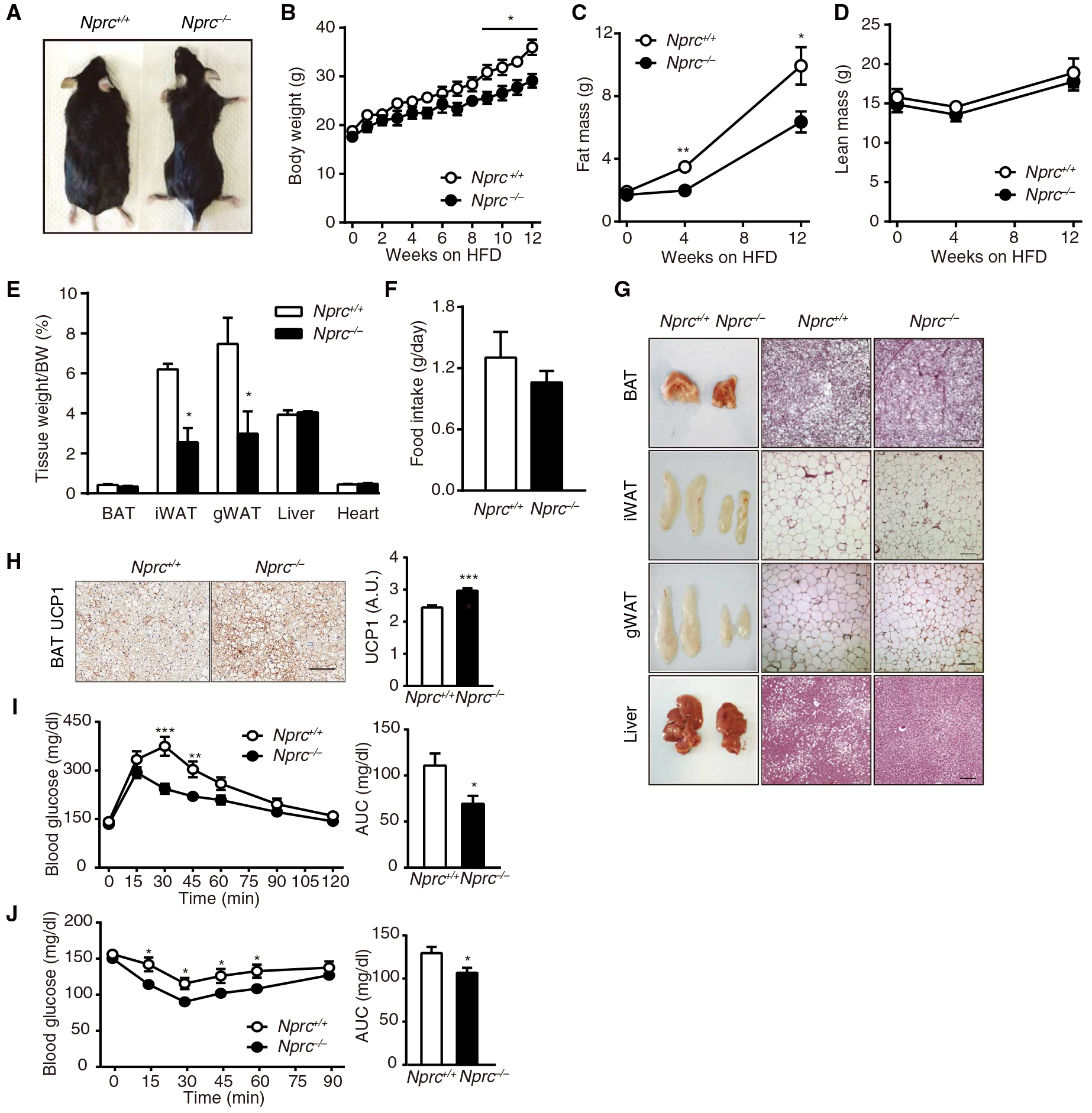
HFD-fed *Nprc*^*−/−*^ mice are leaner and exhibit improved glucose tolerance. (**A**) Image of *Nprc*^*−/−*^ and wild-type (WT) mice after 12weeks on HFD. (**B** to **E**) Body weights (BWs) (B), fat mass (C), lean mass (D), and tissue weights (E)of *Nprc*^*−/−*^ (*n* = 5) and WT (*n* = 6) mice after 12 weeks on HFD. (**F**) Daily food intake of *Nprc*^*−/−*^ (*n* = 3) and WT (*n* = 4) mice on HFD. (**G**) Representative images and hematoxylin and eosin (H&E) staining of BAT, inguinal WAT (iWAT), gWAT, and liver from WT (*n* = 3) and *Nprc*^*−/−*^ (*n* = 3) mice after 12 weeks on HFD. Scale bars, 100 μm. (**H**) Immunostaining and quantification of UCP1 in the BAT of WT (*n* = 2) and *Nprc*^*−/−*^ (*n* = 2) mice after 12 weeks on HFD, as described in Materials and Methods. Scale bar, 200 μm. A.U., arbitrary units. (**I** and **J**) Plasma glucose concentration and area under the curve (AUC) during intraperitoneal glucose tolerance test (GTT) (I) and insulin tolerance test (ITT) (J) of *Nprc*^*−/−*^ (*n* = 12) and WT (*n* = 8) mice after 12 weeks on HFD. *P < 0.05; **P < 0.01; ***P < 0.001, unpaired two-tailed Student’s *t* test.

NPRC was greatly increased at the mRNA and protein levels in the adipose tissue of *Nprc*^*fl/fl*^ mice after 12 weeks on an HFD (Fig. 1, A to C), suggesting that the resulting increase in NPRC may attenuate the ability of NPs to stimulate lipolysis and thermogenesis. *Nprc*^*−/−*^ mice on the HFD gained less weight than wild-type mice, which was primarily due to decreased fat mass (Fig. 2, A to E), without changes in food intake (Fig. 2F). In addition to smaller depots of WAT, *Nprc*^*−/−*^ mice also accumulated less lipid in both the interscapular brown fat (BAT) and the liver (Fig. 2G) and had more UCP1 in the BAT (Fig. 2H). *Nprc*^*−/−*^ mice also showed better glucose and insulin tolerance (Fig. 2, I and J). Consistent with a previous report (23), concentrations of circulating NPs were not altered in *Nprc*^*−/−*^ mice (fig. S1A). Together, these results suggest that *Nprc*^*−/−*^ mice are metabolically healthier than the wild-type mice.

**Fig. 3.**
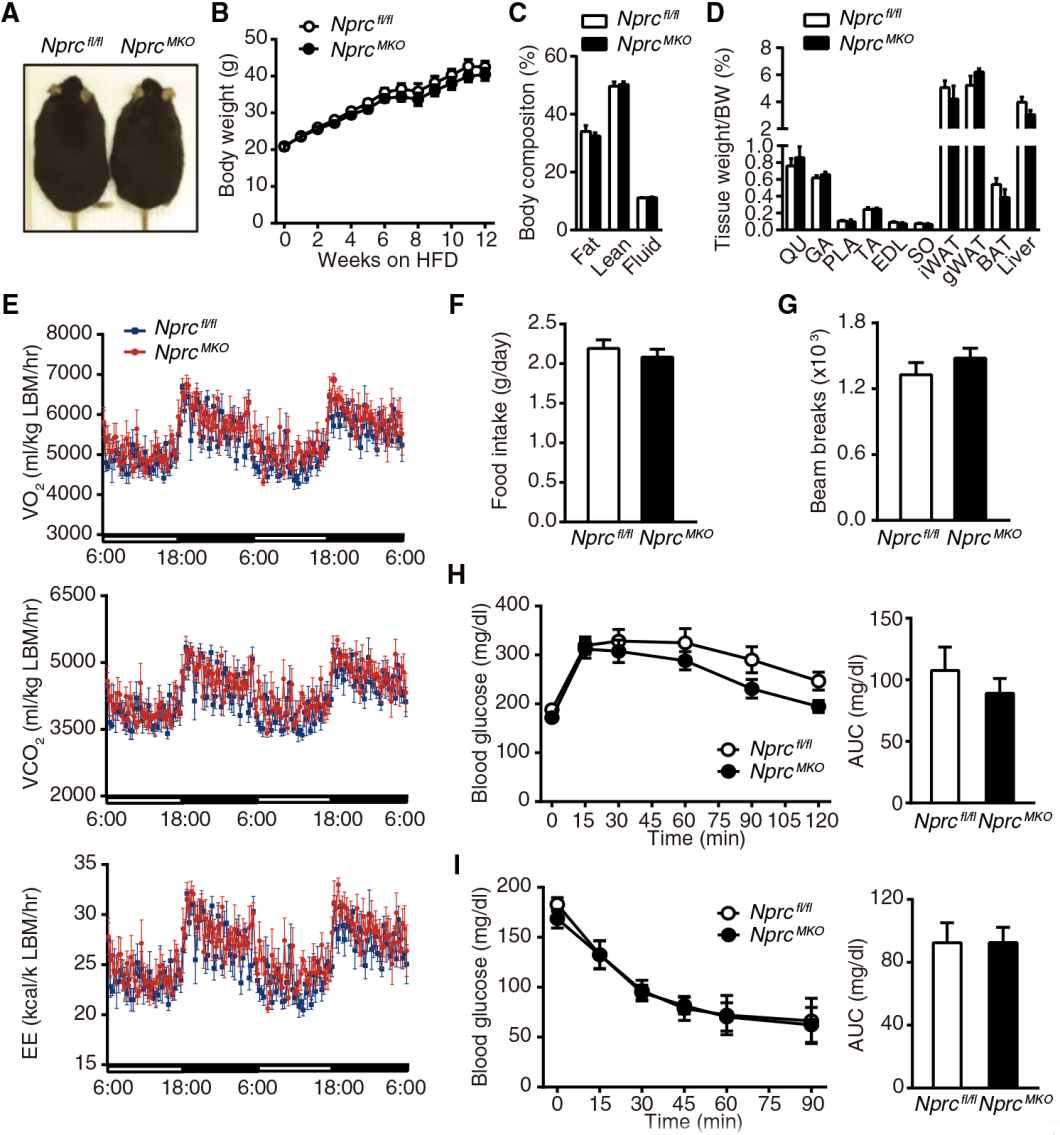
HFD-fed *Nprc* ^*MKO*^ and *Nprc*^*fl/fl*^ mice show comparable body weight, energy expenditure, and glucose tolerance. (**A**) Image of *Nprc*^*MKO*^ and *Nprc*^*fl/fl*^ mice after 12 weeks on HFD. (**B** to **D**) Body weights (B), body composition (C), and tissue weights (D) of *Nprc*^*MKO*^ (*n* = 7) and *Nprc*^*fl/fl*^ (*n* = 6) mice after 12 weeks on HFD. QU, quadriceps; GA, gastrocnemius; PLA, plantaris; TA, tibialis anterior; EDL, extensor digitorum longus; SO, soleus. (**E** to **G**) O_2_ consumption (VO_2_), CO_2_ production (VCO_2_), energy expenditure (EE) (E), food intake (F), and physical activity (G) of *Nprc*^*MKO*^ (*n* = 7) and *Nprc*^*fl/fl*^ (*n* = 6) mice measured by indirect calorimetry using CLAMS (Comprehensive Lab Animal Monitoring System) after 6 weeks on HFD. Data in (E) were normalized to lean body mass (LBM). See fig. S3 for data calculated per body weight and per mouse. (**H** and **I**) Plasma glucose concentration and AUC of *Nprc*^*MKO*^ (*n* = 7) and *Nprc*^*fl/fl*^ (*n* = 6) mice after 12 weeks on HFD during intraperitoneal GTT (H) and ITT (I).

Several studies suggest a role of NPs in regulating skeletal muscle metabolism (24–27). In wild-type mice, the expression of *Nprc* and *Npra* in skeletal muscle was lower than in adipose tissues, but the ratio of *Npra* to *Nprc* was comparable (fig. S2, A and B). To investigate the contribution of adipose tissue and muscle to the leaner phenotype of HFD-fed *Nprc*^*−/−*^ mice, we next generated tissue-specific *Nprc* knockout mice. Skeletal muscle–specific knockout mice (*Nprc*^*MKO*^) were generated by crossing floxed *Nprc* mice (*Nprc*^*fl/fl*^) with *myogenin*-Cre mice (29). *Nprc*^*MKO*^ mice were born in the expected Mendelian and sex ratios. Circulating NP concentrations and blood pressure were comparable between the two genotypes (fig. S1, B and D). *Nprc* expression was efficiently reduced in the skeletal muscle tissues examined, with no changes in expression of *Npra*, as indicated by quantitative real-time polymerase chain reaction (qRT-PCR) (fig. S2, C and D). However, we found that compared to *Nprc*^*fl/fl*^ mice, HFD-fed *Nprc*^*MKO*^ mice had comparable body weight and composition (Fig. 3, A to D), energy expenditure (Fig. 3E and fig. S3, A and B), food consumption (Fig. 3F), physical activity (Fig. 3G), and glucose and insulin tolerance (Fig. 3, H and I). Therefore, contrary to expectation, these results suggest that skeletal muscle did not contribute to the metabolic improvements seen in HFD-fed *Nprc*^*−/−*^ mice.

### HFD-fed *Nprc*^*AKO*^ mice gained less body weight and exhibited higher energy expenditure and improved glucose tolerance

**Fig. 4.**
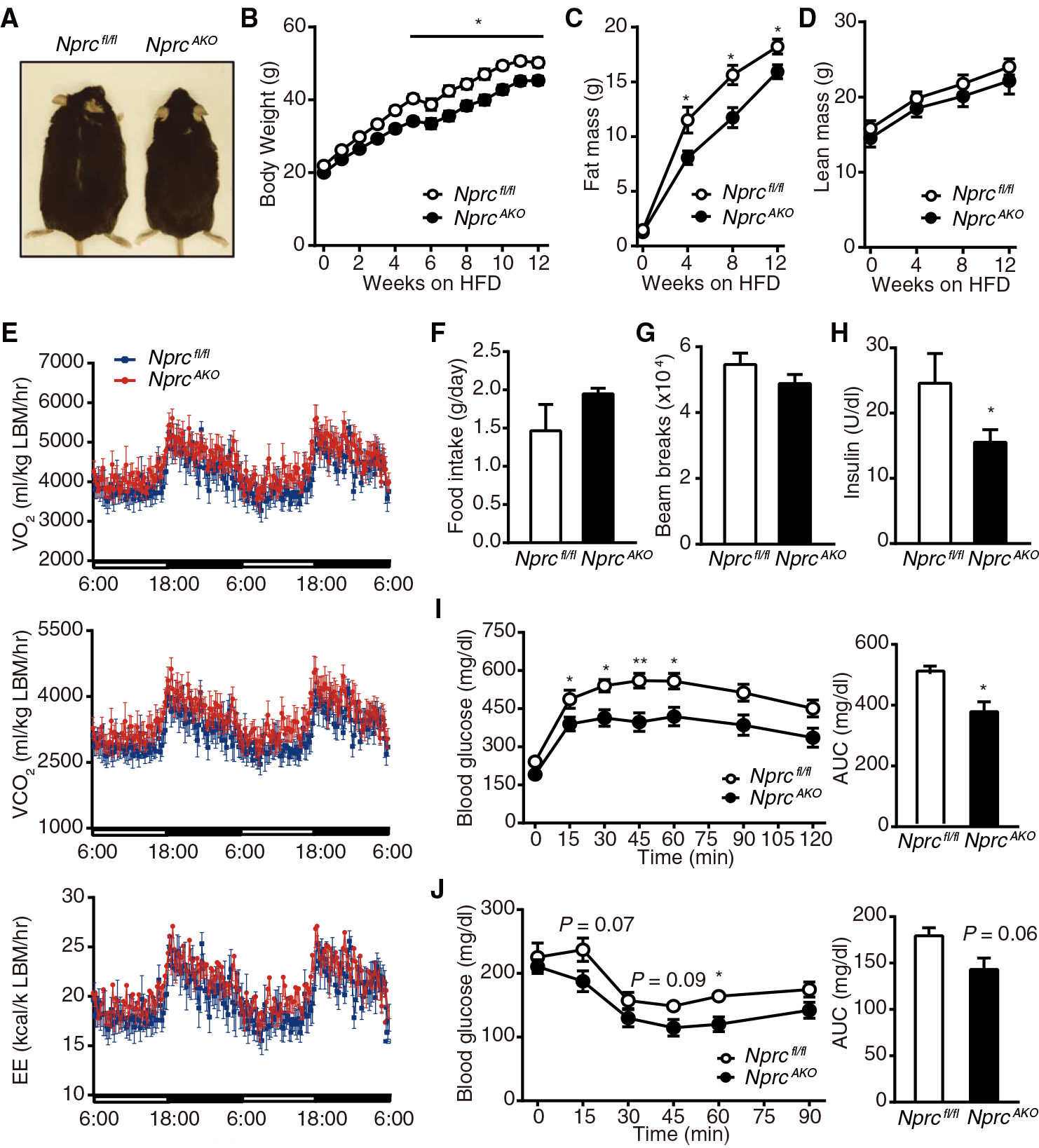
HFD-fed *Nprc*^*AKO*^ mice gain less body weight and exhibit higher energy expenditure and improved glucose tolerance. (**A**) Image of male *Nprc*^*AKO*^ and *Nprc*^*fl/fl*^ mice after 12 weeks on HFD. (**B** to **D**) Body weights (B), fat mass (C), and lean mass (D) of *Nprc*^*AKO*^ (*n* = 14) and *Nprc*^*fl/fl*^ (*n* = 7) mice after 12 weeks on HFD. (**E** to **G**) O_2_ consumption, CO_2_ production, energy expenditure (E), food intake (F), and physical activity (G) of *Nprc*^*AKO*^ (*n* = 8) and *Nprc*^*fl/fl*^ (*n* = 6) mice measured by indirect calorimetry using CLAMS after 5 weeks on HFD. Data in (E) were normalized to lean body mass. See fig. S3 for data calculated per body weight and per mouse. (**H**) Fasting plasma insulin concentration of *Nprc*^*AKO*^ (*n* = 7) and *Nprc*^*fl/fl*^ (*n* = 3) mice after 12 weeks on HFD. (**I** and **J**) Plasma glucose concentration and AUC during intraperitoneal GTT (I) and ITT (J) in *Nprc*^*AKO*^ (*n* = 14) and *Nprc*^*fl/fl*^ (*n* = 7) mice after 12 weeks on HFD. *P < 0.05; **P < 0.01, unpaired two-tailed Student’s *t* test.

Adipocyte-specific *Nprc* knockout mice (*Nprc*^*AKO*^) were generated by crossing the floxed *Nprc* mice with *adiponectin*-Cre mice (30). *Nprc*^*AKO*^ mice were born in the expected Mendelian and sex ratios. Their circulating NP concentrations and blood pressure were comparable to littermate *Nprc*^*fl/fl*^ mice (fig. S1, C and D). NPRC mRNA and protein were absent from the WAT and BAT of the *Nprc*^*AKO*^ mice, but not in the liver, kidney, or heart, whereas there was no change in *Npra* and NPRA in any of the tissues examined (fig. S2, E to G). When fed a HFD, *Nprc*^*AKO*^ mice gained less weight than *Nprc*^*fl/fl*^ mice (Fig. 4, A and B). Total fat mass was reduced in *Nprc*^*AKO*^ mice (Fig. 4C), whereas lean mass was not different between genotypes (Fig. 4D).

Indirect calorimetry analysis showed that oxygen consumption, carbon dioxide production, and energy expenditure increased in HFD-fed *Nprc*^*AKO*^ mice (Fig. 4E and fig. S3, C and D) without alterations in food intake or physical activity (Fig. 4, F and G). After 12 weeks on an HFD, *Nprc*^*AKO*^ mice had a significantly lower fasting plasma insulin concentration compared with *Nprc*^*fl/fl*^ mice (Fig. 4H). GTT revealed that *Nprc*^*AKO*^ mice had lower blood glucose concentrations with a significantly reduced AUC (Fig. 4I). Similarly, ITT showed that the *Nprc*^*AKO*^ mice had improved insulin sensitivity, although the decrease in AUC was not statistically significant (Fig. 4J).

### HFD-fed *Nprc*^*AKO*^ mice showed increased glucose uptake and thermogenesis-related gene expression in the BAT

**Fig. 5.**
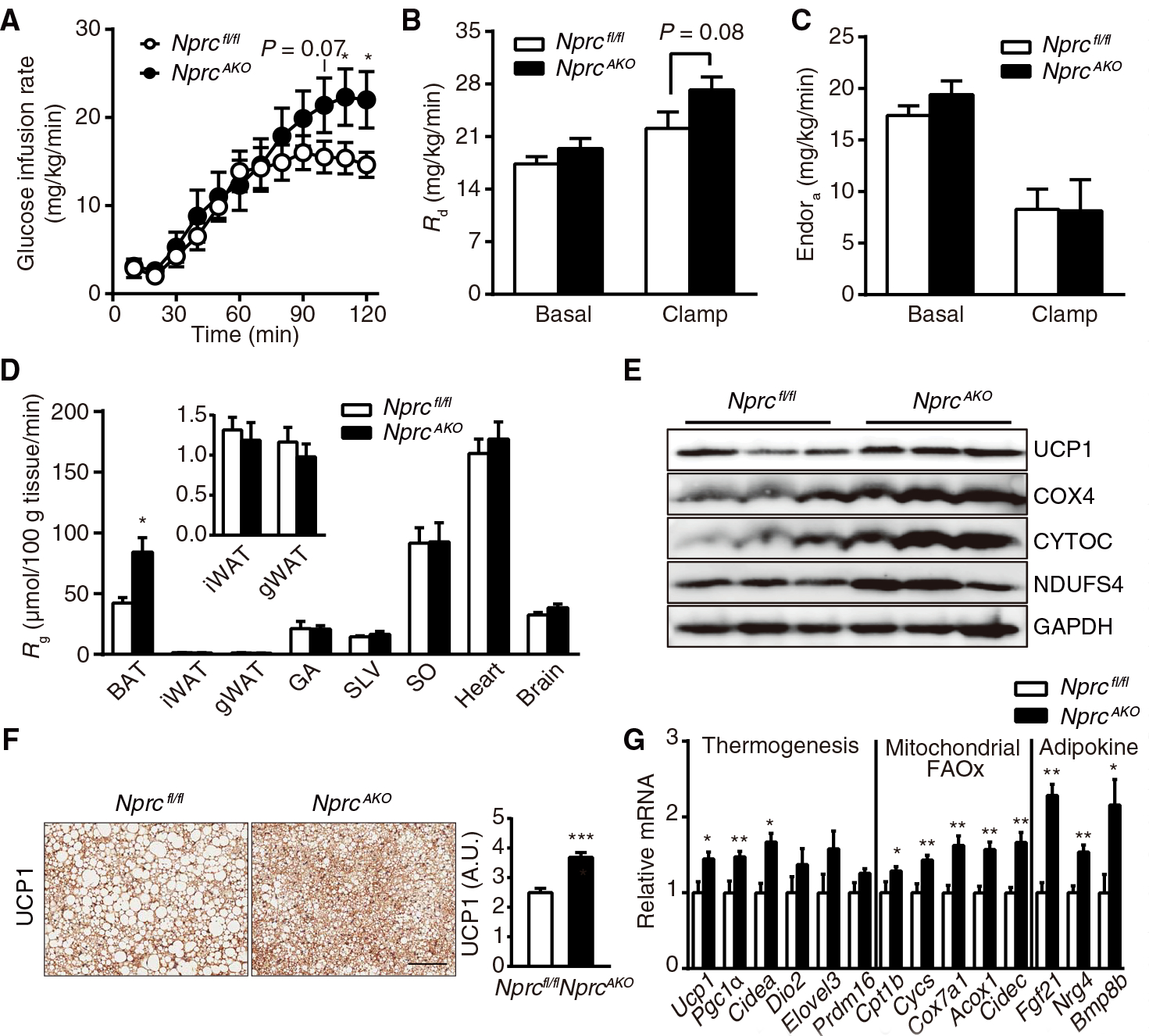
Glucose uptake and expression of thermogenesis markers are increased in the BAT of HFD-fed *Nprc*^*AKO*^ mice. (**A**) Glucose infusion rate of *Nprc*^*AKO*^ (*n* = 10) and *Nprc*^*fl/fl*^ (*n* = 8) mice during a hyperinsulinemic-euglycemic clamp experiment performed at 12 weeks of HFD feeding. (**B** and **C**) Rates of glucose disposal (*R*_d_) (B) and endogenous glucose appearance (Endo*R*_a_) (C) in *Nprc*^*AKO*^ (*n* = 10) and *Nprc*^*fl/fl*^ (*n* = 8) mice under basal and clamp states. (**D**) Rate of glucose uptake (*R*_g_) in the BAT, iWAT, gWAT, gastrocnemius (GA), superficial vastus lateralis (SVL), soleus (SO), heart, and brain of *Nprc*^*AKO*^ (*n* = 10) and *Nprc*^*fl/fl*^ (*n* = 8) mice at the end of the clamp experiment. (**E**) Western blot analysis of thermogenic and mitochondrial proteins in the BAT of *Nprc*^*AKO*^ and *Nprc*^*fl/fl*^ mice after 12 weeks on HFD. Blots are representative of three independent experiments. COX4, cytochrome c oxidase subunit 4; CYTOC, cytochrome c; NDUFS4, NADH (reduced form of nicotinamide adenine dinucleotide) dehydrogenase ubiquinone iron-sulfur protein 4; GAPDH, glyceraldehyde-3-phosphate dehydrogenase. (**F**) Immunostaining and quantification of UCP1 in the BAT of *Nprc*^*AKO*^ (*n* = 2) and *Nprc*^*fl/fl*^ (*n* = 2) mice after 12 weeks on HFD, as described in Materials and Methods. Scale bar, 200 μm. (**G**) qRT-PCR for the expression of genes coding thermogenic, mitochondrial, fatty acid oxidation (FAOx) markers, and BAT-derived adipokines in the BAT of *Nprc*^*AKO*^ (*n* = 14) and *Nprc*^*fl/fl*^ (*n* = 7) mice after 12 weeks on HFD. Data were relative to *36B4*. *P < 0.05; **P < 0.01; ***P < 0.001. *P* values of glucose infusion rate were obtained by one-tailed Student’s *t* test. *P* values of *R*_d_, *R*_a_, *R*_g_, and relative mRNA expression were calculated using unpaired two-tailed Student’s *t* tests.

We next examined insulin action in conscious, unrestrained mice by hyperinsulinemic-euglycemic clamp. *Nprc*^*AKO*^ mice had a higher glucose infusion rate than *Nprc*^*fl/fl*^ mice (Fig. 5A), indicating enhanced insulin sensitivity. In line with this finding, there was a statistically insignificant increase in the rate of endogenous glucose disappearance (*R*_d_) in *Nprc*^*AKO*^ mice (Fig. 5B), suggesting increased glucose disposal. In both genotypes, glucose appearance rate (*R*_a_) was comparably suppressed by the insulin clamp, illustrating that hepatic insulin action was not different between genotypes (Fig. 5C). Furthermore, there was a significantly higher rate of glucose uptake (*R*_g_) into BAT than *Nprc*^*fl/fl*^ mice but not into other tissues examined (Fig. 5D).

Because the most substantial difference in glucose uptake in the clamp studies between the genotypes was observed in the BAT, we examined whether this difference was associated with higher thermogenic activity in the BAT of *Nprc*^*AKO*^ mice. Compared to *Nprc*^*fl/fl*^ control mice, UCP1 was more abundant in the BAT of *Nprc*^*AKO*^ mice (Fig. 5, E and F). Consistent with this finding, the transcription of a battery of key thermogenic genes (for example, *Pgc1*α and *Cidea*), mitochondrial genes (for example, *Cpt1b*, *Cycs*, and *Cox7*α*1*), and fatty acid oxidation genes (for example, *Acox1* and *Cidec*) was also higher in the BAT from *Nprc*^*AKO*^ mice compared to *Nprc*^*fl/fl*^ mice (Fig. 5G). In addition, the transcription of several genes encoding BAT-derived adipokines, including *Fgf21*, *Nrg4*, and *Bmp8b*, also increased in the BAT of *Nprc*^*AKO*^ mice (Fig. 5G). Although the absence of NPRC in iWAT and gWAT might be expected to favor increased expression of thermogenesis-related genes in those tissues (16), the two genotypes did not show differences for the expression of any of these genes except for *Cidec* (fig. S4, A and B). We also saw no histological evidence of “beige” or UCP1-positive cells (fig. S4C). Thus, contrary to expectation, deletion of *Nprc* promoted thermogenesis in the BAT but not in iWAT or gWAT for *Nprc*^*AKO*^ mice, at least in the setting of diet-induced obesity.

### *Nprc* deficiency in adipocytes results in a healthy redistribution of lipid storage and prevents inflammation in adipose tissue

**Fig. 6.**
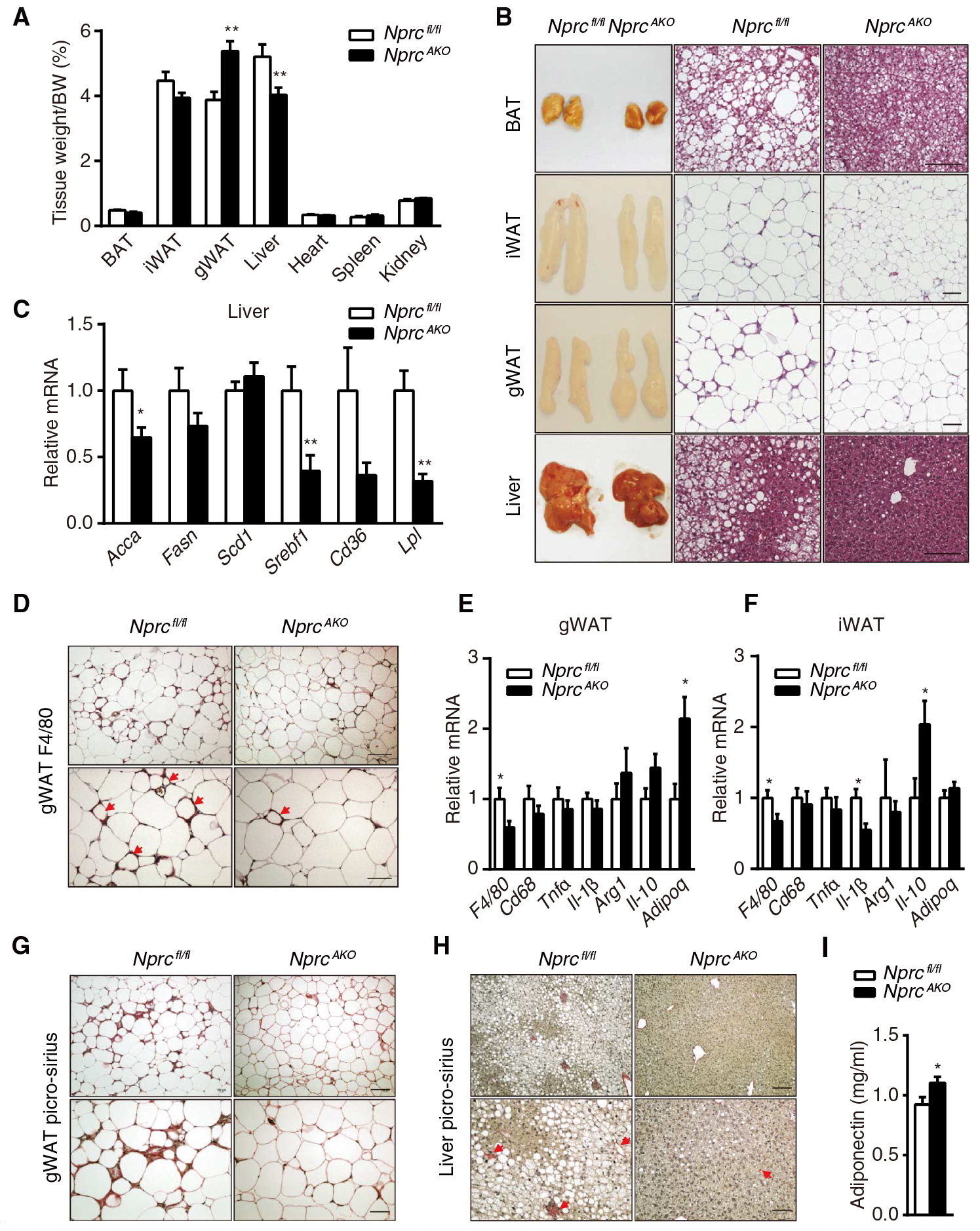
HFD-fed *Nprc*^*AKO*^ mice are protected from hepatic steatosis and adipose tissue inflammation. (**A**) Tissue weight of *Nprc*^*AKO*^ (*n* = 14) and *Nprc*^*fl/fl*^ (*n* = 7) mice after 12 weeks on HFD. (**B**) Representative images and H&E staining of BAT, iWAT, gWAT, and liver from *Nprc*^*fl/fl*^ (*n* = 3) and *Nprc*^*AKO*^ (*n* = 3) mice. Scale bars, 100 mm. (**C**) qRT-PCR for the expression of markers of de novo lipogenesis and fatty acid uptake in the liver of HFD-fed *Nprc*^*AKO*^ (*n* = 8) and *Nprc*^*fl/fl*^ (*n* = 7) mice. Data were relative to *36B4*. (**D**) Immunostaining of F4/80 in gWAT sections from *Nprc*^*fl/fl*^ (*n* = 2) and *Nprc*^*AKO*^ (*n* = 2) mice. Crown-like structures are indicated by arrows. Scale bars, 100 μm (top) and 50 μm (bottom). (**E** and **F**) qRT-PCR for the expression of genes coding macrophage markers and inflammatory cytokines in gWAT (E) and iWAT (F) of HFD-fed *Nprc*^*AKO*^ (*n* = 14) and *Nprc*^*fl/fl*^ (*n* = 7) mice. Data were relative to *36B4*. (**G** and **H**) Picro-sirius staining of gWAT (G) and liver (H) sections from *Nprc*^*fl/fl*^ (*n* = 2) and *Nprc*^*AKO*^ (*n* = 2) mice. Collagen fibrils were stained red (H; arrows). Scale bars, 100 μm (top) and 50 μm (bottom). (**I**) Plasma concentrations of adiponectin of HFD-fed *Nprc*^*AKO*^ (*n* = 8) and *Nprc*^*fl/fl*^ (*n* = 7) mice. *P < 0.05; **P < 0.01, unpaired two-tailed Student’s *t* test.

Upon dissection, gross examination of tissues showed that despite an overall reduced body fat (Fig. 4C), the mass of the iWAT fat pad was not different between genotypes (Fig. 6A). However, *Nprc*^*AKO*^ mice had increased gWAT mass, whereas liver weight was significantly reduced (Fig. 6A). The size of the adipocytes in the iWAT de pots from *Nprc*^*AKO*^ mice was smaller than those from *Nprc*^*fl/fl*^ mice (fig. S5, A and B). The BAT of *Nprc*^*AKO*^ mice did not change in overall mass but had a slightly darker complexion and smaller lipid droplets compared with that of *Nprc*^*fl/fl*^ mice (Fig. 6, A and B). In addition, the livers of *Nprc*^*AKO*^ mice were essentially devoid of steatosis (Fig. 6, A and B), which was associated with reduced expression of lipogenic and fatty acid uptake genes (such as *Lpl*, *Acca*, and *Srebf1*) (Fig. 6C). In the gWAT depot of *Nprc*^*fl/fl*^ mice, there was a high density of crown-like structures (Fig. 6B), indicative of inflammatory macrophage infiltration into the adipose tissue that is associated with insulin resistance (31). In contrast, few crown-like structures were detected in *Nprc*^*AKO*^ gWAT. Immunohistochemical staining for the macrophage-specific marker F4/80 confirmed the robust presence of macrophages in the gWAT from *Nprc*^*fl/fl*^ mice but not in that from *Nprc*^*AKO*^ mice (Fig. 6D). qRT-PCR analysis further confirmed that *F4/80* expression was significantly reduced in gWAT of *Nprc*^*AKO*^ mice (Fig. 6E), whereas genes related to proinflamma-tory M1 macrophage activation (such as *Cd68*, *Tnf*α, and *Il-1*β) were not significantly different (Fig. 6E). The expression of genes encoding anti-inflammatory M2 macrophage markers (such as *Arg1* and *Il-10*) increased in *Nprc*^*AKO*^ mice in a nonstatistically significant manner. Although crown-like structures were not detected in iWAT of either genotype, there was nevertheless a significant reduction in F4/80 (Fig. 6F). In *Nprc*^*AKO*^ mice, there was also a significant increase in *Il-10* expression (Fig. 6F), which encodes an antifibrotic and anti-inflammatory cytokine (32). Adipose inflammation is closely associated with fibrosis (33). Compared to *Nprc*^*fl/fl*^ mice, fibrosis was reduced in the gWAT of *Nprc*^*AKO*^ mice, as indicated by Picro-sirius staining (Fig. 6G). Similarly, in line with the amelioration of hepatic steatosis, focal liver fibrosis was also reduced in *Nprc*^*AKO*^ mice (Fig. 6H). Moreover, adiponectin, an insulin-sensitizing hormone that promotes macrophage polarization toward an anti-inflammation phenotype (34, 35), significantly increased in gWAT at the mRNA level (Fig. 6E) and in the plasma (Fig. 6I) of *Nprc*^*AKO*^ mice.

**Fig. 7.**
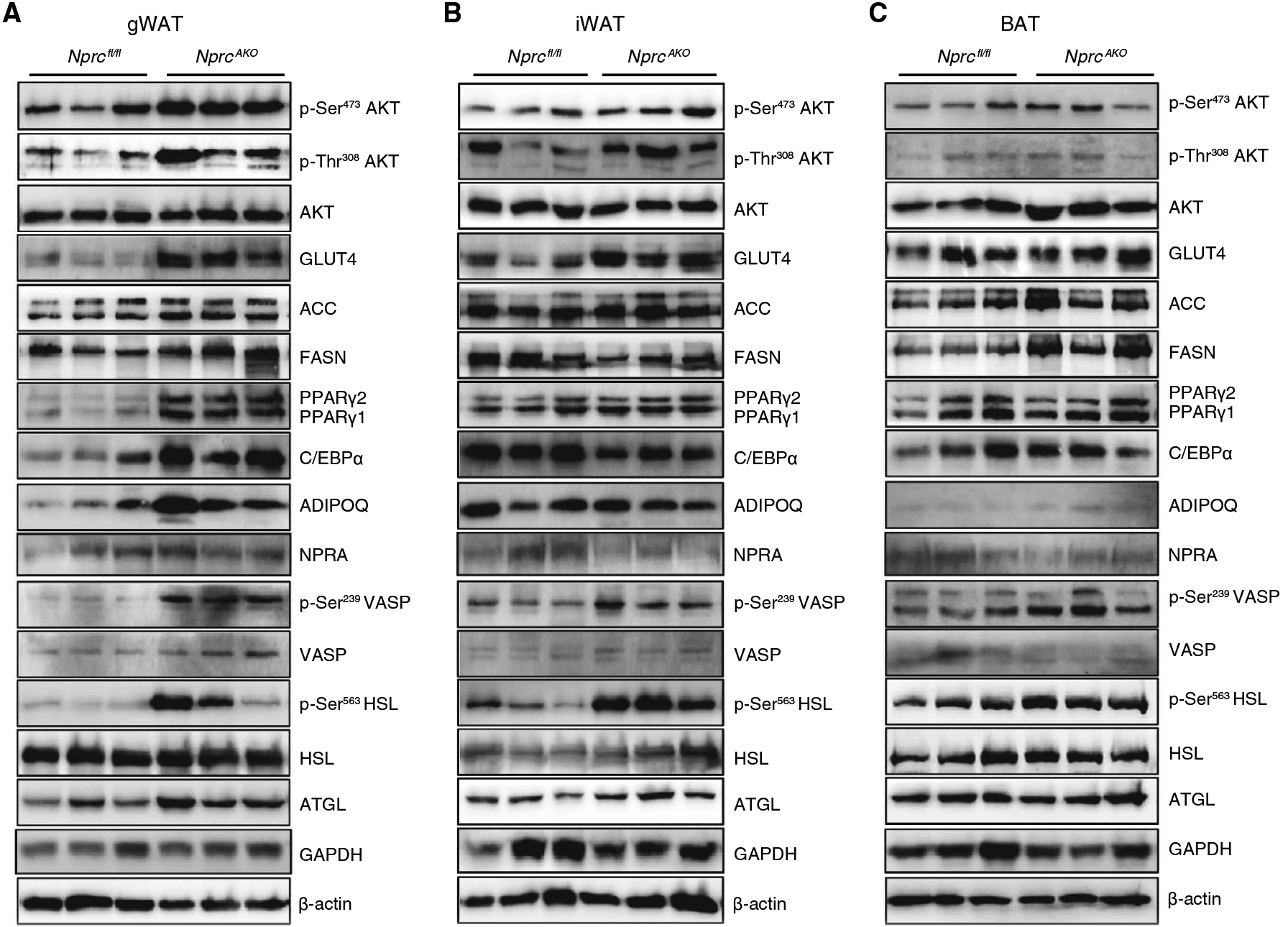
Insulin signaling, de novo lipogenesis, adipogenesis, lipolysis, and PKG activity in adipose tissues of HFD-fed *Nprc*^*AKO*^ mice. (**A** to **C**) Western blotting analysis for proteins involved in insulin signaling (p-Ser^473^ AKT, p-Thr^308^ AKT, and GLUT4), de novo lipogenesis (ACC and FASN), adipogenesis (PPARγ, C/EBPα, and ADIPOQ), PKG activity (p-Ser^239^ VASP), and lipolysis (p-Ser^563^ HSL and ATGL) performed on lysates from gWAT (A), iWAT (B), and BAT (C) of *Nprc*^*AKO*^ and *Nprc*^*fl/fl*^ mice after 12 weeks on HFD. p, phosphorylated. Blots are representative of three independent experiments.

*Nprc*^*AKO*^ mice on an HFD gained less body weight and showed improved insulin sensitivity, despite a larger gWAT depot than in the *Nprc*^*fl/fl*^ control mice. Basal insulin signaling was increased in gWAT of the *Nprc*^*AKO*^ mice, as evidenced by increased AKT phosphorylation and glucose transporter type 4 (GLUT4) protein (Fig. 7A). In addition, the abundance of acyl–coenzyme A carboxylase (ACC) and fatty acid synthase (FASN), markers of de novo lipogenesis, was also higher in the gWAT of *Nprc*^*AKO*^ mice. The abundance of key transcriptional regulatorsofadipogenesis, peroxisome proliferator–activated receptor α (PPARα), and CCAAT/enhancer binding protein (C/EBPα), were also higher in gWAT tissue samples from *Nprc*^*AKO*^ mice (Fig. 7A). Furthermore, the abundance of adiponectin also increased at the protein level in gWAT of *Nprc*^*AKO*^ mice (Fig. 7A). Active adipogenesis and lipogenesis in the gWAT depot of *Nprc*^*AKO*^ mice are consistent with the increased mass of this depot.

The amount of NPRA did not change, but PKG activity, as indicated by the phosphorylation of vasodilator-stimulated phosphoprotein (VASP), was greater in the gWAT of *Nprc*^*AKO*^ mice (Fig. 7A). Consistent with increased PKG activity, there was an increase in phosphorylated hormone-sensitive lipase (HSL) and total adipose triglyceride lipase (ATGL), suggesting that in the *Nprc*^*AKO*^ gWAT tissue there was also a higher degree of lipolysis in addition to increased lipogenesis and triglyceride deposition. In the iWAT of the *Nprc*^*AKO*^ mice, basal insulin signaling activity increased (Fig. 7B). However, there was still an increase in phosphorylated VASP and phosphorylated HSL, suggesting higher PKG signaling and lipolysis. In the BAT of the *Nprc*^*AKO*^ mice, there were no apparent changes in markers of insulin signaling, de novo lipogenesis, or adipogenesis but a moderate increase in phosphorylated VASP and phosphorylated HSL (Fig. 7C).

The increased PKG activity in *Nprc*^*AKO*^ adipose tissues was further confirmed in vitro using primary adipocytes differentiated from the stromal vascular fraction of the iWAT depots from the two genotypes. Cells from both genotypes differentiated into adipocytes to a similar extent (fig. S6A). When stimulated by a cocktail containing ANP and BNP, *Nprc*-deficient adipocytes showed greater PKG activity, as indicated by phosphorylation of VASP, and higher lipolytic activity (fig. S6, B to D), indicating that *Nprc*-deficient adipocytes were more responsive to NP stimulation. We used human embryonic kidney (HEK) 293 cells that stably express NPRA [also known as HEK293-GCA cells (36)] to further test, in a controlled way, the role of NPRC in the ANP dose-response curve. The presence of NPRC resulted in a shift of the cGMP dose-response curve to the right, compared to HEK293-GCA cells without NPRC (fig. S6, E and F). Together, these data indicated that NPRC deletion from adipose tissue increased the potency of ANP, thereby increasing PKG signaling and leading to an improvement in the metabolic phenotypes.

## DISCUSSION

The link between obesity and the cardiac NPs originates with reports by Sarzani *et al*. showing that receptors for ANP and BNP are present in adipose tissue (14) and that obese human subjects often have substantially higher amounts of the NP clearance receptor NPRC in adipose tissue and lower circulating NPs (18, 37). An increase in NPRC relative to the signaling receptor NPRA renders the tissue less responsive to NPs (fig. S6, E and F). The high amount of NPRC in the adipose tissue of obese subjects gave rise to the notion that adipose tissue might be a “sink” for circulating NPs, which could contribute to the hypertension that is often associated with obesity (18, 38). It is noteworthy also that lower plasma NP concentrations in obese human subjects could also be the result of reduced secretion of NPs because obesity is also associated with lower concentrations of N-terminal pro-BNP, a precursor fragment that is not cleared by NPRC (39).

ANP stimulates lipolysis in cultured human adipocytes through increases in cGMP and PKG activity, in a manner parallel to β-adrenergic receptors (βARs) and cyclic adenosine 5′-monophosphate/protein kinase A [reviewed in (40)]. NPRA activation also leads to uncoupled respiration in brown adipocytes and increases browning of WAT, again in a parallel pathway to the βARs (41). Because a growing body of evidence places the NP system at the center of “cardiometabolic” disease (42), including the adipocyte response to early pathological stress on the heart (43), we studied their tissue-specific effects by manipulating NPRC abundance.

Mice with naturally occurring mutations that disrupt the expression of the *Nprc* gene are longer due to delayed bone ossification and have very little body fat, giving rise to their original names of “longjohn” and “strigosus” (28). We have shown that the WAT and BAT of *Nprc*^*−/−*^ mice have smaller lipid droplets and an increased ability to respond to NPs for lipolysis (16). Here, we showed that *Nprc*^*−/−*^ mice were also protected from HFD-induced obesity and glucose intolerance. This metabolic benefit was retained in *Nprc*^*AKO*^ mice but not in *Nprc*^*MKO*^ mice. These findings indicated that the role of NPRC in regulating NP-mediated metabolism during HFD-feeding conditions largely depended on the adipose tissue but not on the skeletal muscle. This finding is consistent with previous findings that the improvement of insulin sensitivity in obese and diabetic subjects is associated with an increased NPRA/NPRC ratio in adipose tissue but not in skeletal muscle (19). Because NPs promote oxidative metabolism in human skeletal muscle (17, 27), the role of NPRC in skeletal muscle metabolism in the *Nprc*^*MKO*^ mice may need to be investigated under other physiological settings, such as exercise performance or in response to direct NP infusions, as has been done in humans (25).

We showed here that targeted *Nprc* deficiency in adipose tissue increased oxygen consumption and carbon dioxide production without altering food intake and physical activity. Notably, UCP1 and other mitochondrial proteins increased in the BAT of *Nprc*^*AKO*^ mice, suggesting higher thermogenic activity. These findings are consistent with a previous study, wherein transgenic overexpression of either BNP or PKG decreases body weight and increases the expression of genes involved in thermogenesis in the BAT of mice consuming either a chow diet or an HFD (26). An important observation from our findings that needs to be further studied is that the expression of *Fgf21*, *Nrg4*, and *Bmp8b*, which encode BAT-enriched adipokines, increased in the BAT of *Nprc*^*AKO*^ mice. FGF21 (fibroblast growth factor 21) regulates energy expenditure, glucose homeostasis, and insulin sensitivity, potentially through the action of adiponectin (44, 45). Neuregulin 4 (NRG4) is a brown fat–enriched secreted factor that preserves metabolic homeostasis through attenuation of hepatic lipogenesis (46). Bone morphogenetic protein 8B (BMP8B) increases BAT thermogenesis through both central and peripheral actions (47). It is possible that one of the effects of NP signaling in the BAT is the synthesis and release of these “batokines.” Consequently, the increased production of these batokines in *Nprc*^*AKO*^ mice may also contribute to the overall improved metabolic profile of these mice in an endocrine and/or paracrine manner. The reduced liver fibrosis that we observed in *Nprc*^*AKO*^ mice is reminiscent of previous findings that continuous ANP intravenous infusion or transgenic BNP overexpression prevents liver fibrosis (48, 49). Because our *Nprc*^*AKO*^ mice did not show differences in NPRC or NPRA abundance in the livers compared with *Nprc*^*fl/fl*^ mice (fig. S2, E to G), we favor the interpretation that the absence of steatosis was the primary reason for reduced fibrosis. Moreover, whether NRG4 is responsible for the reduced hepatic steatosis and fibrosis of *Nprc*^*AKO*^ mice is still to be explored.

Here, we found no indications of “beiging” in the WAT of either *Nprc*^*−/−*^ or *Nprc*^*AKO*^ mice when fed an HFD. This finding may be due to additional unidentified factors induced by HFD feeding that could weaken the adipose browning program driven by NP. In addition to increased thermogenesis in the BAT, the gWAT mass of HFD-fed *Nprc*^*AKO*^ mice unexpectedly increased, as did de novo lipogenesis and adipogenesis. PKG promotes brown adipocyte differentiation and mitochondrial biogenesis (50). Whether the cGMP pathway is involved in adipogenesis in WAT depots deserves further exploration (51). Moreover, the gWAT of HFD-fed *Nprc*^*AKO*^ mice was metabolically healthier. This finding is reminiscent of a previous study showing that the capacity to expand adipose tissue can be associated with greater insulin sensitivity (52).

Adipose tissue inflammation links obesity to insulin resistance (53). Along with improved insulin sensitivity, we found that in *Nprc*^*AKO*^ mice, the gWAT did not contain macrophage infiltration, as seen in the gWAT of *Nprc*^*AKO*^ mice. It is not clear whether the loss of NPRC from adipocytes is responsible for the reduced inflammation, which, in turn, improved insulin sensitivity or vice versa. PKG activity was increased, potentially through NP/NPRA signaling, in the visceral fat of *Nprc*^*AKO*^ mice, and adiponectin in both plasma and gWAT increased accordingly. Although the effects on adipogenesis, lipogenesis, and inflammation together might be responsible for the improved insulin sensitivity in *Nprc*^*AKO*^ mice, the mechanistic connection between the enhanced NP signaling and improved insulin sensitivity is yet to be investigated.

In conclusion, our results highlight a key role of cardiac NPs and their signaling in adipose tissue to regulate systemic energy expenditure and glucose homeostasis. Adipose-specific deletion of *Nprc* protects against diet-induced obesity and insulin resistance by activating thermogenesis and glucose uptake in the BAT, increasing lipolysis in subcutaneous iWAT, and alleviating inflammation while promoting tissue expansion in visceral gWAT. These various mechanisms of metabolic adaptation suggest the versatile functions of NP signaling in the control of energy metabolism in different adipose depots. These studies demonstrate that attenuation of NPRC activity in adipose tissue has the potential to be a therapeutic mechanism for controlling metabolic disease.

## MATERIALS AND METHODS

### Reagents and antibodies

The protease inhibitor cocktail (cOmplete Mini) and a phosphatase inhibitor cocktail (PhosSTOP) were obtained from Roche Diagnostics. The following antibodies were obtained from Cell Signaling Technology: Cox4 (4844), PPARγ (2435), C/EBPα (8178), p-Ser^239^ VASP (3114), total VASP (3112), p-Ser^473^ AKT (4060), p-Thr^308^ AKT (9275), total AKT (9272), p-Ser^563^ HSL (4139), total HSL (4107), ATGL (2439), ADIPOQ (2789), GLUT4 (2213), ACC (3676), FASN (3180), and β-actin (4967). Other antibodies used include NPRC (NBP1–31365, Novus Biologicals), NPRA (NBP1–31333, Novus Biologicals), NDUFS4 (PA5–21677, Pierce Biotechnology), UCP1 (ab23841, Abcam), and GAPDH (sc25788, Santa Cruz Biotechnology) and CYTOC (sc13156, Santa Cruz Biotechnology). Secondary antibodies against rabbit immunoglobulin G (IgG) (A3687) and mouse IgG (A3562), both conjugated with alkaline phosphatase, were from Sigma-Aldrich.

### Animal experiments

*Nprc*^*−/−*^ mice (backcrossed to C57BL/6J for at least eight times) were a gift from N. Maeda and O. Smithies (Department of Pathology and Laboratory Medicine, University of North Carolina at Chapel Hill, Chapel Hill, NC, with the Mutant Mouse Regional Resource Center). Mice with a floxed *Nprc* allele were generated for this study by the Knockout Mouse Project at the University of California, Davis. *Myogenin*-Cre mice were a gift from E. Olson of University of Texas (UT) Southwestern Medical Center and bred to generate mice with skeletal muscle–specific disruption of the *Nprc* gene. *Adiponectin*-Cre mice (JAX-010803) were purchased from the Jackson Laboratory and bred to generate mice with adipose-specific disruption of the *Nprc* gene. Tail DNA genotyping and qRT-PCR of lysate from different tissues were used to confirm the specific deletion of *Nprc* in skeletal muscles and adipose tissues. Mice were kept under a 12-hour light/12-hour dark cycle at constant temperature (23°C) with unlimited access to food and water. A 60% HFD (D12492, Research Diets) was provided starting at 7 weeks of age and maintained for 12 weeks, during which time body weight and body composition were monitored. After the 12-week feeding period, mice were euthanized, and tissues were carefully dissected, weighed, and either immediately frozen in liquid nitrogen or processed for histology. All animal studies were approved by the Institutional Animal Care and Use Committe of Sanford Burnham Prebys Medical Discovery Institute in accordance with the eighth edition of the National Institutes of Health (NIH) *Guide for the Care and Use of Laboratory Animals*.

### Primary adipocyte cell culture, oil red O staining, and glycerol release

Primary adipocytes were prepared from iWAT and differentiated, as previously described (54). For oil red O staining, differentiated adipocytes were fixed with 3.7% formaldehyde for 10 min, stained with 0.5% oil red O for 1 hour at room temperature, and then washed with water. Glycerol release was evaluated with the free glycerol reagent from Sigma-Aldrich (F6428) according to the manufacturer’s instruction.

### Indirect calorimetry

After 5 weeks (*Nprc*^*AKO*^) or 6 weeks (*Nprc*^*MKO*^) on HFD, mice were housed and monitored seperately in open-circuit Oxymax chambers (Columbus Instruments) with free access to HFD and drinking water for 72 hours. Oxygen comsumption, carbon dioxide production, and physical activity were monitored at 15- to 20-min intervals. Food and water intake was also recorded. Data from the first 24 hours were discarded because the mice adapted to the new housing.

### GTT and ITT and hyperinsulinemic-euglycemic clamps

During the last week of HFD, mice were fasted for 5 hours, glucose (1 g/kg) was injected intraperitoneally, and blood glucose concentrations were measured through tail vein at 0, 15, 30, 45, 60, 90, and 120 min with Bayer CONTOUR blood glucose monitoring system. For the ITT, insulin (0.8 U/kg) was administered intraperitoneally, and blood glucose concentrations were measured at 0, 15, 30, 60, and 90 min. The AUC was calculated from the baseline and divided by the period of time. Hyperinsulinemic-euglycemic clamps were performed as previously described (55).

### Plasma insulin, adiponectin, and measurement of ANP and BNP

Blood samples were collected by cardiac puncture after a 5-hour fast using heparin anticoagulation tube. Enzyme-linked immunosorbent assay kit was used to detect plasma concentrations of insulin (Mercodia), adiponectin (AdipoGen), and ANP and BNP (Phoenix Pharmaceuticals) according to the respective manufacturer’s instructions.

### Tissue histology and microscopy

Adipose tissues and liver were fixed with 4% paraformaldehyde in phosphate-buffered saline (PBS), dehydrated, embedded in paraffin, and cut into 5-μm sections. Sections were stained with H&E and examined under bright-field microscopy with a Nikon 80i. Adipocyte sizes were quantified using ImageJ software with the “Adipocyte” application tool. The UCP1 and F4/80 staining was performed on the Leica BOND RX automated system. Sections were deparaffinized, rehydrated, heated for antigen retrieval, and then incubated with UCP1 antibody (1:400 dilution; ab10983, Abcam) at room temperature for 2 hours and F4/80 antibody (1:50 dilution; HM1066, Hycult Biotech) for 1 hour. After washing in the Leica BOND wash buffer, slides were incubated with Rat Probe, followed by incubation with Rat-on-Mouse AP-Polymer (RT518, Biocare Medical) according to the manufacturer’s protocol. Slides were then reacted with the chromogen from the Bond Polymer Refine Detection kit (DS9800, Leica Biosystems) for UCP1 staining and the Bond Polymer Refine Red Detection kit (DS9390, Leica Biosystems) for F4/80 staining. Slides were counterstained with hematoxylin, and coverslip was added. UCP1 staining was quantified by analyzing five randomly selected fields from two sections of individual mice with a positive pixel count application tool (Aperio, Leica Biosystems). Picro-sirius staining was performed on paraffin sections of gWAT and livers using a Picrosirius Red Stain kit according to the manufacturer’s instructions (24901, Polysciences).

### RNA isolation and qRT-PCR

RNA was extracted from tissues with TRIzol reagent (Invitrogen) and purified by RNA Mini columns (Qiagen). Reverse transcription (Applied Biology) and SYBR green qRT-PCR (Life Technologies) were performed according to the manufacturer’s protocols. Target primer sequences are presented in table S1. qRT-PCR results were analyzed by ΔΔ*C*_t_ method, normalized to the internal control gene *36B4*, and expressed as mean ± SEM.

### Protein extraction and Western blotting

Protein was extracted from tissues, as previously described (56). For Western blotting analysis, 40 mg of protein, unless otherwise indicated, was resolved by 10% SDS–polyacrylamide gel electrophoresis, transferred to nitrocellulose membranes (Bio-Rad), and incubated overnight at 4°C with specific primary antibodies. Secondary antibodies conjugated with alkaline phosphatase were used for specific protein detection.

### Mitochondrial DNA isolation and quantitative PCR

Mitochondrial DNA (mtDNA) was extracted from the skeletal muscle, as previously described (57). mtDNA and nuclear DNA were determined by evaluating the abundance of the DNA encoding NADH dehydrogenase subunit 1 and lipoprotein lipase by ΔΔ*C*_t_ methods, as previously described (57).

### cGMP dose-response assay

HEK293 cells that were stably transfected with the green fluorescent protein–tagged *NPR1* plasmid (also known as HEK293-GCA cells) were a gift from J. Burnett (36). HEK293-GCA cells were transfected with NPRC–YFP (yellow fluorescent protein) or YFP plasmids by polyethylenimine when reaching 80 to 90% confluency and replated into gelatin-coated six-well plates for treatments. Three days after transfection, cells were placed into serum-free media for 16 hours. The non-selective phosphodiesterase inhibitor 3-isobutyl-1-methylxanthine (IBMX; 0.5 μM) and the phosphodiesterase 9 (cGMP-specific and IBMX-insensitive phosphodiesterase) inhibitor BAY73–6691 (50 nM) were added to the cells for 1 hour before stimulation with 0 to 3000 nM ANP. cGMP production was stopped after 15 min of ANP treatment by placing cells on ice and washing with ice-cold PBS. Cells were lysed into 300 μl of PBS by four cycles of freeze-thaw-vortex method. The concentrations of cGMP were measured with a homemade competitive enzyme immunoassay kit. Briefly, protein G–coated plates (15133, Pierce) were washed with wash buffer (PBS + 1% Tween 20), coated with 100 μl per well of antibody against cGMP (1:1000; ab836, Abcam) in 0.05 M carbonate bicarbonate buffer (pH 9.6) for 2 hours, and blocked with blocking buffer (5% bovine serum albumin in PBS) for 1 hour at room temperature (RT). Fifty microliters of cell lysates per well were incubated with 50 μl of cGMP–horseradish peroxidase antibody (1:10,000; M01058, GenScript) and diluted in blocking buffer for 2 hours at RT. Plates were washed with washing buffer and incubated with 100 μl per well of TMB reagent (BioFX Laboratories) for 15 min at RT. The reaction was stopped by adding 100 μl per well of 0.25 M H_2_SO_4_. Optical density at 450 nm was read, and the data were plotted as linear B/B0% versus log cGMP concentration using a four-parameter logistic fit.

### In vivo pressure hemodynamics

In vivo pressure hemodynamics was performed as described (58). Briefly, male mice (3 to 4 months old) were injected with etomidate (10 mg/kg intraperitoneally) and then placed in an induction chamber filled with 3.5% isoflurane mixed with 100% oxygen. Sedated mice were then intubated with a 20-gauge catheter sleeve, placed atop a heated surgical board, and connected to a small animal ventilator (model SAR-830/P; CWE Inc.). Respiration was set to 14- to 18-cm H_2_O at a rate between 140 and 160 breaths per minute, and isoflurane levels were adjusted using a vaporizer to attain a surgical plane of anesthesia (2 to 3%). A small area of skin in the neck region was removed, the sublingual salivary glands were gently separated, and a section (15 to 20 mm) of the right common carotid was isolated caudally from the bifurcation of the internal and external carotid. To aid in vessel cutdown and pressure catheter insertion, a 4–0 suture was tied at the bifurcation, a small vascular clamp was placed as far caudally as possible, and a second 4–0 suture was placed between the two. The vessel was then cut down, and a 1.4-F pressure catheter was inserted (model SPR-671, Millar Instruments) and advanced to near the aortic valve. Pressure traces were recorded using LabChart 8.0 (ADInstruments) software, and isoflurane was reduced to 1.5%. A rectal probe was then inserted to monitor core body temperature, which was held at 37 ± 1°C. After 10 min, which allowed the animal ample time to recuperate from surgery, consecutive pressure tracings from at least 30 to 60 s worth of stable recordings were analyzed using the built-in tools of the LabChart software.

### Statistical analysis

All data are means ± SEM. Unpaired two-tailed Student’s *t* tests or two-way analysis of variance (ANOVA) followed by post hoc tests with Bonferroni’s correction for multiple comparisons tests was used to examine differences between groups, as appropriate. Glucose infusion rate was analyzed by one-tailed Student’s *t* test. Statistical significance was defined as *P* < 0.05.

## Supplementary Material

Supplemental Material Fig. S1. Blood pressures and circulating NP concentrations of *Nprc*^*−/−*^, *Nprc*^*MKO*^, and *Nprc*^*AKO*^ mice.
Fig. S2. Expression of *Nprc* and *Npra* in skeletal muscle and adipose tissue.
Fig. S3. Alternative representation of CLAMS indirect calorimetry data of HFD-fed *Nprc*^*MKO*^ and *Nprc*^*AKO*^ mice.
Fig. S4. HFD-fed *Nprc*^*AKO*^ and *Nprc*^*fl/fl*^ mice show comparable expression of thermogenic-related genes in gWAT and iWAT.
Fig. S5. Adipocyte size distribution in iWAT and gWAT of HFD-fed *Nprc*^*fl/fl*^ and *Nprc*^*AKO*^ mice.
Fig. S6. NPRC deficiency enhances NP signaling.
Table S1. PCR primer sequences.
