# Supplementary material for: Enhancing natriuretic peptide signaling in adipose tissue, but not in muscle, protects against diet-induced obesity and insulin resistance
Source: Sci Signal. Author manuscript; Available in PMC 2020 Aug 11. (PMC7418652; doi:10.1126/scisignal.aam6870)
Supplement: Supplemental Material — Fig. S1. Blood pressures and circulating NP concentrations of Nprc−/−, NprcMKO, and NprcAKO mice. Fig. S2. Expression of Nprc and Npra in skeletal muscle and adipose tissue. Fig. S3. Alternative representation of CLAMS indirect calorimetry data of HFD-fed NprcMKO and NprcAKO mice. Fig. S4. HFD-fed NprcAKO and Nprcfl/fl mice show comparable expression of thermogenic-related genes in gWAT and iWAT. Fig. S5. Adipocyte size distribution in iWAT and gWAT of HFD-fed Nprcfl/fl and NprcAKO mice. Fig. S6. NPRC deficiency enhances NP signaling. Table S1. PCR primer sequences. [file NIHMS1612540-supplement-Supplemental_Material.pdf]

Science Signaling
AAAS

www.sciencesignaling.org/cgi/content/full/10/489/eaam6870/DC1

Supplementary Materials for

Enhancing natriuretic peptide signaling in adipose tissue, but not in muscle, protects against diet-induced obesity and insulin resistance

Wei Wu, Fubiao Shi, Dianxin Liu, Ryan P. Ceddia, Robert Gaffin, Wan Wei, Huafeng Fang, E. Douglas Lewandowski, Sheila Collins*

*Corresponding author. Email: scollins@sbpdiscovery.org

Published 25 July 2017, *Sci. Signal.* **10**, eaam6870 (2017)
DOI: 10.1126/scisignal.aam6870

This PDF file includes:

Fig. S1. Blood pressures and circulating NP concentrations of $Nprc^{-/-}$, $Nprc^{MKO}$, and $Nprc^{AKO}$ mice.
Fig. S2. Expression of *Nprc* and *Npra* in skeletal muscle and adipose tissue.
Fig. S3. Alternative representation of CLAMS indirect calorimetry data of HFD-fed $Nprc^{MKO}$ and $Nprc^{AKO}$ mice.
Fig. S4. HFD-fed $Nprc^{AKO}$ and $Nprc^{fl/fl}$ mice show comparable expression of thermogenic-related genes in gWAT and iWAT.
Fig. S5. Adipocyte size distribution in iWAT and gWAT of HFD-fed $Nprc^{fl/fl}$ and $Nprc^{AKO}$ mice.
Fig. S6. NPRC deficiency enhances NP signaling.
Table S1. PCR primer sequences.

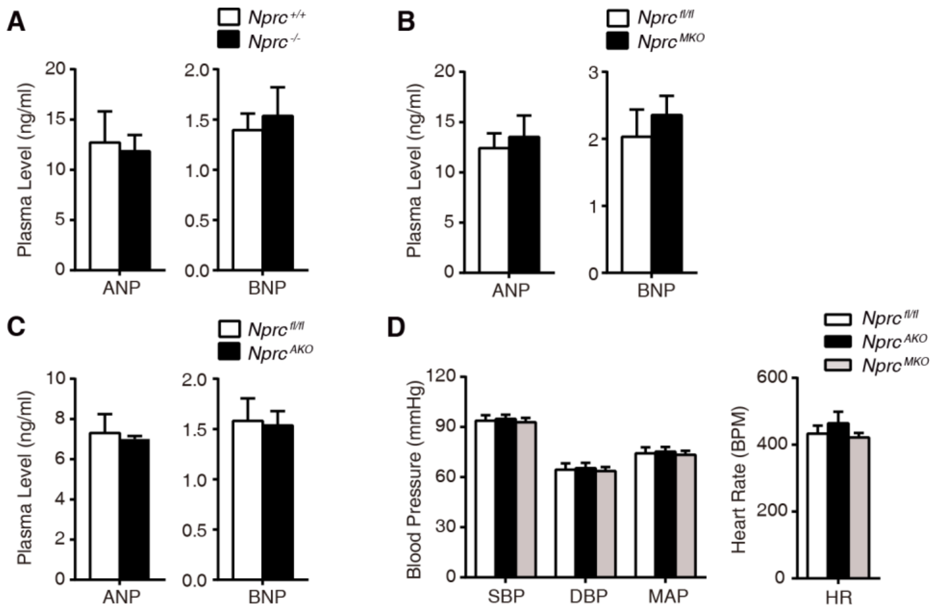

Fig. S1. Blood pressures and circulating NP concentrations of *Nprc*$^{-/-}$, *Nprc*^{MKO}, and *Nprc*^{AKO} mice. (A to C) Circulating ANP (left) and BNP (right) concentrations in (A) *Nprc*$^{-/-}$, (B) *Nprc*^{MKO} and (C) *Nprc*^{AKO} mice (n=5 for each group). The variation of plasma NP concentration between these three lines may be due to differences in the duration of storage in the freezer. (D) Systolic blood pressure (SBP), diastolic blood pressure (DBP), mean artery pressure (MAP) and heart rate (HR) of *Nprc*^{AKO} and *Nprc*^{MKO} mice (n=5 for each group) examined by *in vivo* pressure hemodynamics as described in Materials and Methods. MAP=2/3*DBP+1/3*SBP.

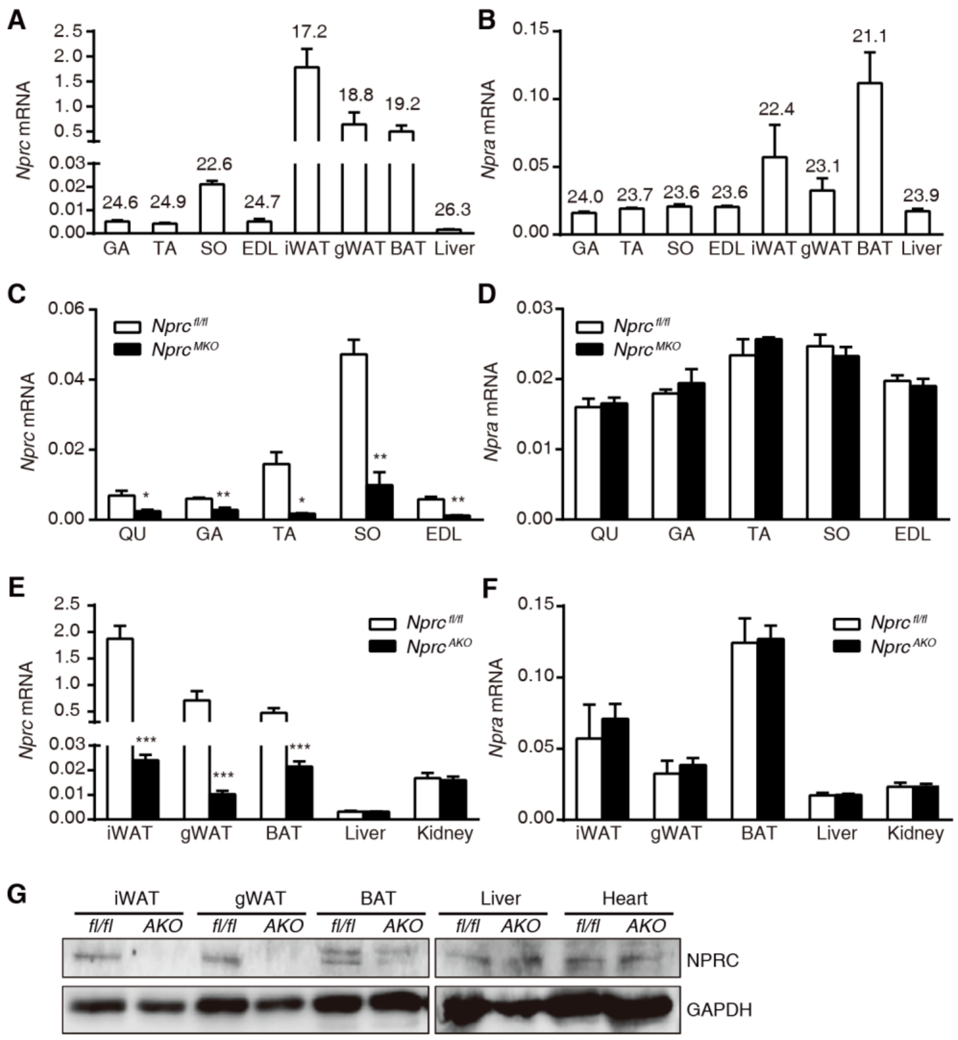

Fig. S2. Expression of *Nprc* and *Npra* in skeletal muscle and adipose tissue. (A to B) qRT-PCR for the expression of *Nprc* (A) and *Npra* (B) relative to *36B4* in gastrocnemius (GA), tibialis anterior (TA), soleus (SO), extensor digitorum longus (EDL), iWAT, gWAT, BAT and liver of *Nprc*$^{fl/fl}$ mice (n=4-5). The average of Ct values for each tissue is as indicated. (C to D) qRT-PCR for the expression of *Nprc* (C) and *Npra* (D) relative to *36B4* in quadriceps (QU), gastrocnemius (GA), tibialis anterior (TA), soleus (SO) and extensor digitorum longus (EDL) of *Nprc*^{MKO} (n=3) and *Nprc*$^{fl/fl}$ (n=4) mice. (E to F) qRT-PCR for the expression of *Nprc* (E) and *Npra* (F) relative to *36B4* in iWAT, gWAT, BAT, liver and kidney of *Nprc*^{AKO} (n=14) and *Nprc*$^{fl/fl}$ (n=6) mice. (G) Western blotting analysis for NPRC on lysates from iWAT, gWAT, BAT, liver and heart of *Nprc*^{AKO} and *Nprc*$^{fl/fl}$ mice. Blots are representative of three independent experiments. *, $p<0.05$; **, $p<0.01$; ***, $p<0.001$. P-values were calculated using unpaired two-tailed Student's t-tests.

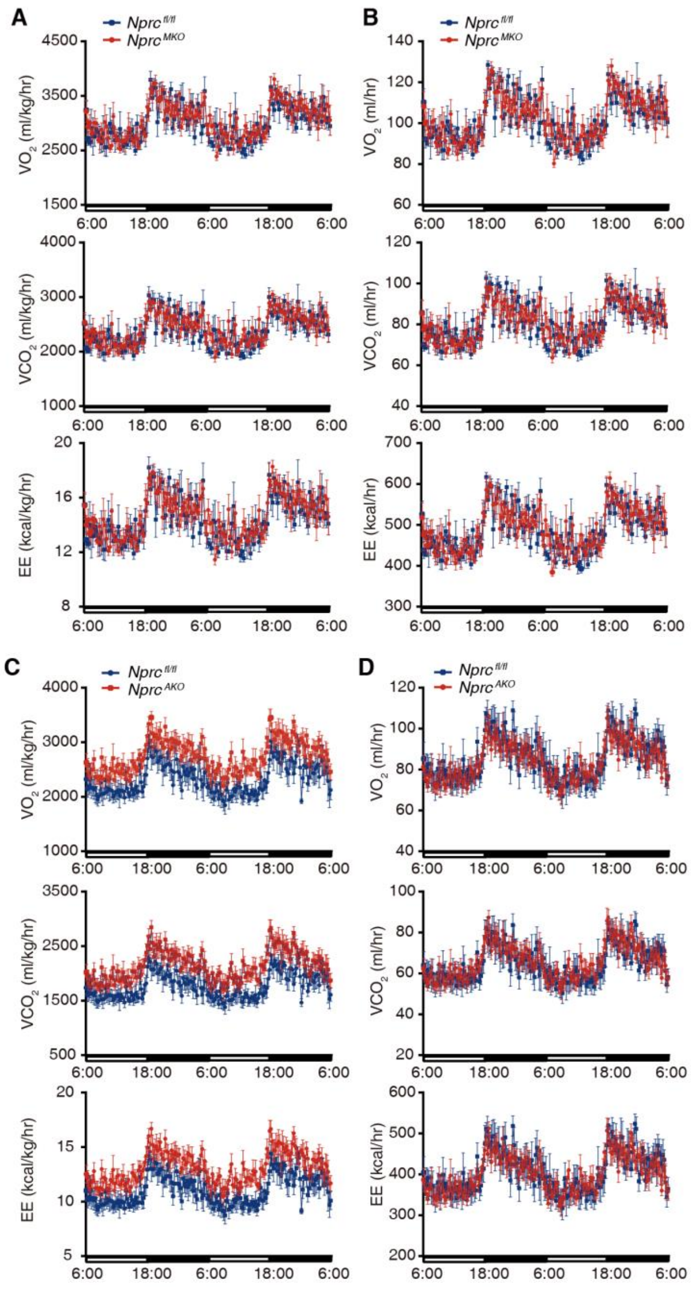

Fig. S3. Alternative representation of CLAMS indirect calorimetry data of HFD-fed $\boldsymbol{Nprc^{MKO}}$ **and** $\boldsymbol{Nprc^{AKO}}$ **mice.** (A to B) O_2 consumption (VO_2), CO_2 production (VCO_2) rates and energy expenditure (EE) of $Nprc^{MKO}$ (n=6) and $Nprc^{fl/fl}$ (n=5) mice measured by indirect calorimetry using CLAMS after 6 weeks on HFD. Data were normalized to total body mass (A) or per mouse (B) (C to D) O_2 consumption (VO_2), CO_2 production (VCO_2) rates and energy expenditure (EE) of $Nprc^{AKO}$ (n=8) and $Nprc^{fl/fl}$ (n=6) mice measured by indirect calorimetry using CLAMS after 5 weeks on HFD. Data were normalized to total body mass (C) or per mouse (D). P values were calculated using two-way ANOVA.

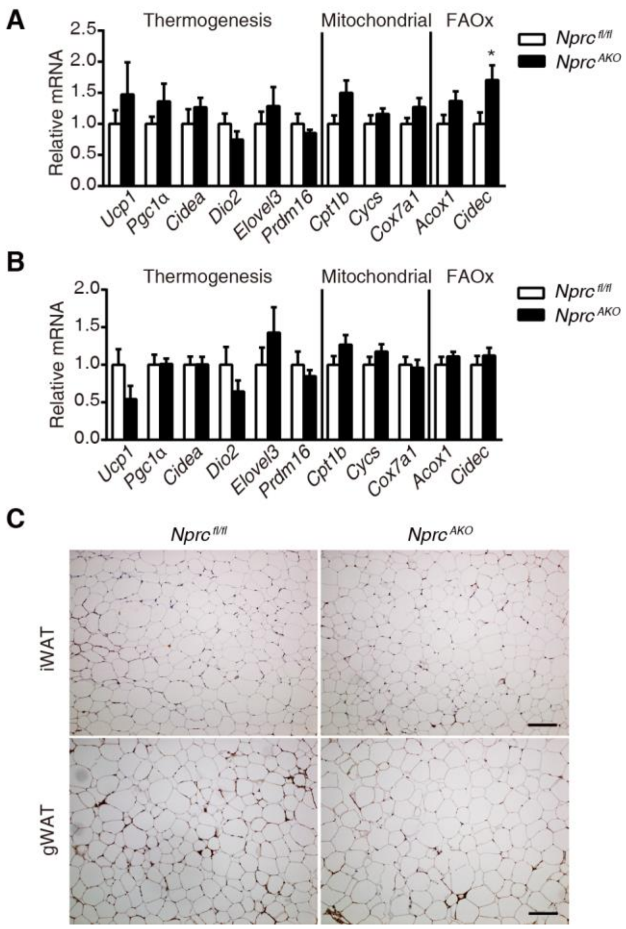

Fig. S4. HFD-fed *Nprc*^{AKO} and *Nprc*$^{fl/fl}$ mice show comparable expression of thermogenic-related genes in gWAT and iWAT. (A to B) qRT-PCR for the expression of genes encoding thermogenic, mitochondrial and fatty acid oxidation (FAOx) markers in gWAT (A) and iWAT (B) of *Nprc*^{AKO} (n=14) and *Nprc*$^{fl/fl}$ (n=7) mice after 12 weeks on HFD. *, $p<0.05$. P-values were calculated using unpaired two-tailed Student's t-tests. (C) Immunostaining of UCP1 in iWAT and gWAT of *Nprc*^{AKO} (n=2) and *Nprc*$^{fl/fl}$ (n=2) mice after 12 weeks on HFD. Scale bar: 200μm.

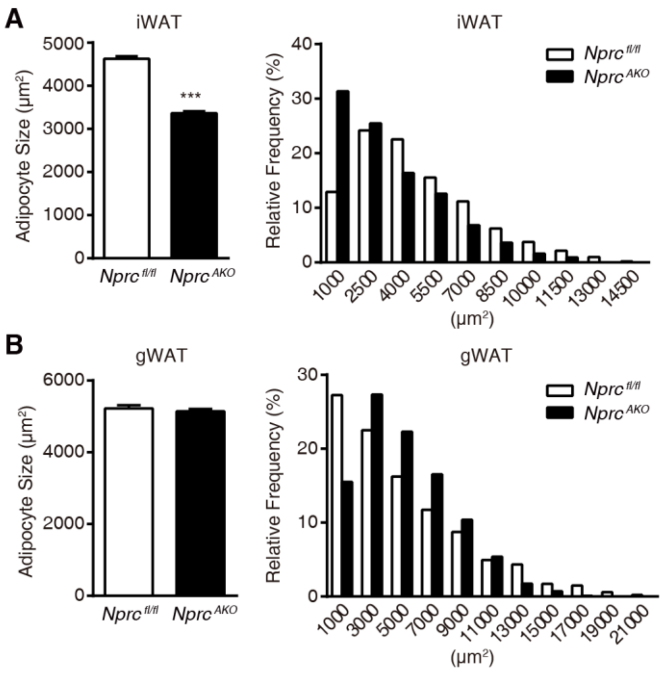

Fig. S5. Adipocyte size distribution in iWAT and gWAT of HFD-fed *Nprc*$^{fl/fl}$ and *Nprc*^{AKO} mice. (A to B) Average adipocyte areas (left) and the distribution of adipocyte areas (right) in (A) iWAT and (B) gWAT. ***, $p<0.001$. P values were calculated using unpaired two-tailed Student's t-tests.

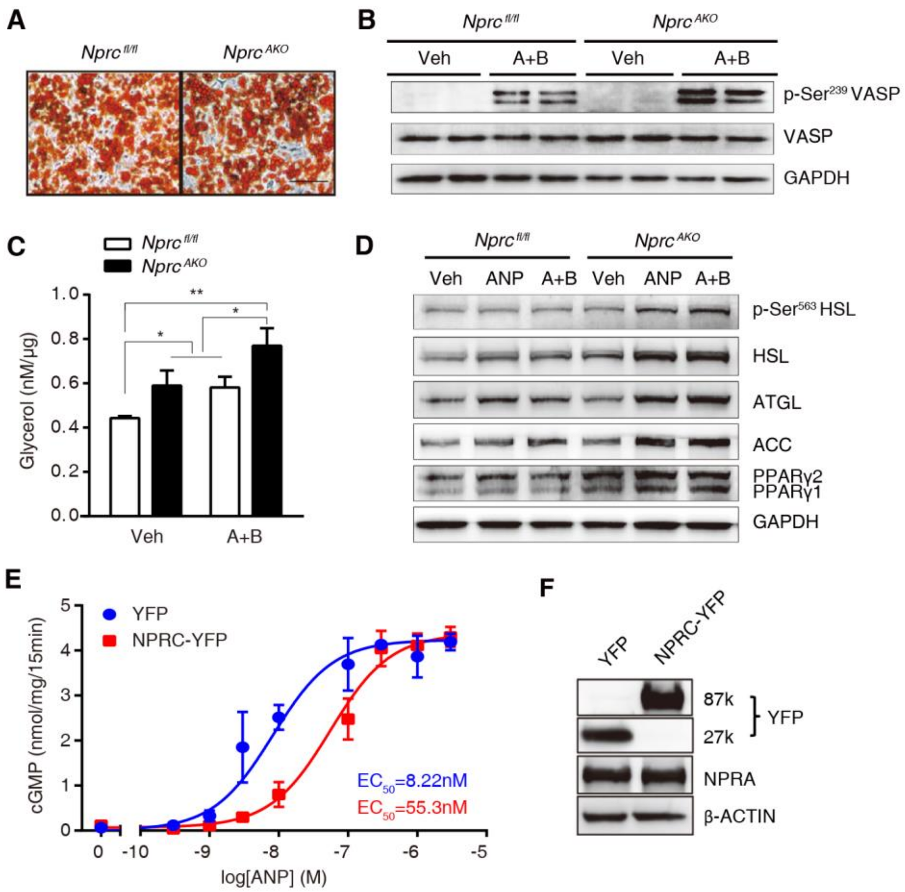

Fig. S6. NPRC deficiency enhances NP signaling. (A) Oil Red O staining of adipocytes differentiated from the stromal vascular fraction of iWAT of *Nprc*^{AKO} and *Nprc*$^{fl/fl}$ mice. Scale bar: 100μm. iWATs from 3-5 mice of each genotype were pooled together for each experiment. Images were representative of three independent experiments. (B) Western blot analysis of PKG activity (p-Ser^{239} VASP) performed on lysates from in primary adipocytes treated with vehicle (Veh) or ANP and BNP (200nM each, A+B) for 30 min. Blots are representative of three independent experiments. (C) Glycerol release into the media by primary adipocytes treated with vehicle (Veh) or ANP and BNP (200nM each, A+B) for 20 hrs. *, p<0.05; **, p<0.01; n=4 independent biological repeats for each experiment, data is representative of three independent experiments. P values were calculated using unpaired two-tailed Student's t-tests. These values do not take into account the possibility of intracellular glycerol being re-esterifed by glycerol kinase. (D) Western blot analysis of proteins involved in lipolysis (p-Ser^{563} HSL, and ATGL), lipogenesis (ACC) and adipogenesis (PPARγ) performed on the lysates from primary adipocytes treated with vehicle (Veh), ANP (200nM) or ANP and BNP (200nM each, A+B) for 20 hrs. Blots are representative of three independent experiments. (E) cGMP dose-response of HEK293-GCA cells transfected either with YFP or NPRC-YFP plasmid. Graph is representative (means ± SD) of three independent experiments. (F) Western blot analysis of NPRC-YFP, YFP and NPRA with the lysate of corresponding samples in E. Blots are representative of three independent experiments.

Table S1. PCR primer sequences.

Genes	Forward (5'-3')	Reverse (5'-3')
36B4	GATGCCCAGGGAAGACAG	ACAATGAAGCATTTTGGATAATCA
Nprc	AGCTGGCTACAGCAAGAAGG	CGGCGATACCTTCAAATGTC
Npra	TGGAGACACAGTCAACACAGC	CGAAGACAAGTGGATCCTGAG
Ucp1	GGCCTCTACGACTCAGTCCA	TAAGCCGGCTGAGATCTTGT
Pgc1α	GAAAGGGCCAAACAGAGAGA	GTAAATCACACGGCGCTCTT
Cidea	GTCTGCAAGCAACCAAAGAA	ATTGAGACAGCCGAGGAAGT
Dio2	CGCTCCAAGTCCACTCGCGG	CGGCCCCATCAGCGGTCTTC
Elovl3	ACTTCGAGACGTTTCAGGACTTA	GACGACCACTATGAGAAATGAGC
Prdm16	CAGCACGGTGAAGCCATTC	GCGTGCATCCGCTTGTG
Cpt1b	GAGTGACTGGTGGGAAGAATATG	GCTGCTTGCACATTTGTGTT
Cycs	ACCAAATCTCCACGGTCTGTTCGG	GGTGATGCCTTTGTTCTTGTTGGC
Cox7α1	CGAAGAGGGGAGGTGACTC	AGCCTGGGAGACCCGTAG
Acox1	GCCCAACTGTGAC	GCCAGGACTATCG
Cidec	GGCTCACAGCTTGGAGGA	CTCCACGATTGTGCCATCT
Accα	CCACACTGAACCGGAAATCT	ATTTGTCGTAGTGGCCGTTC
Fasn	TTCCAAGACGAAAATGATGC	AATTGTGGGATCAGGAGAGC
Scd1	CCTGCGGATCTTCCTTATCATT	GATCTCGGGCCCATTCG
Srebf1	ATCCAGGTCAGCTTGTTTGCGATG	TGGACTACTAGTGTTGGCCTGCTT
F4/80	CTTTGGCTATGGGCTTCCAGTC	GCAAGGAGGACAGAGTTTATCGTG
Cd68	TCCAAGATCCTCCACTGTTG	ATTTGAATTTGGGCTTGGAG
Tnfα	ACGGCATGGATCTCAAAGAC	AGATAGCAAATCGGCTGACG
Il-1β	TGCAGAGTTCCCCAACTGGTACATC	GTGCTGCCTAATGTCCCCTTGAATC
Arg1	CTCCAAGCCAAAGTCCTTAGAG	AGGAGCTGTCATTAGGGACATC
Il-10	GCTCTTACTGACTGGCATGAG	CGCAGCTCTAGGAGCATGTG
Adipoq	GCAGGCATCCCAGGACATC	GCGATACATATAAGCGGCTTCT
Fgf21	TGGGGGTCAAGTCCGGCAGA	TCCAGGAGACTTTCTGGACTGCGG
Ngr4	TCTGTCGGCAGCTTTCGT	CTGAAGGTGGCCCTTCCT
Bmp8b	CTGTATGAACTCCACCAACCAC	GGGGATGATATCTGGCTTCA
Cd36	TGGCCAAGCTATTGCGACAT	AGGCATTGGCTGGAAGAACA
Lpl	AGCAGCAAGACCTTCGTGG	TCTCTCTTGTACAGGGCGGC
ND1(mt)	CCCATTCGCGTTATCTT	AAGTTGATCGTAACGGAAGC
LPL (nu)	GGATGGACGGTAAGAGTGATTC	ATCCAAGGGTAGCAGACAGGT
